# Motivations Associated with Food Choices among Adults from Urban Setting

**DOI:** 10.3390/foods12193546

**Published:** 2023-09-24

**Authors:** Ana Ilić, Ivana Rumbak, Dina Dizdarić, Marijana Matek Sarić, Irena Colić Barić, Raquel Pinho Ferreira Guiné

**Affiliations:** 1 Department of Food Quality Control, Faculty of Food Technology and Biotechnology, University of Zagreb, Pierottijeva 6, 10000 Zagreb, Croatia; ailic@pbf.hr (A.I.); icolic@pbf.hr (I.C.B.); 2 Department of Health Studies, University of Zadar, Splitska 1, 23000 Zadar, Croatia; marsaric@unizd.hr; 3 CI&DETS, Polytechnic Institute of Viseu, Av. José Maria Vale de Andrade, 3504-510 Viseu, Portugal; raquelguine@esav.ipv.pt

**Keywords:** eating behavior, eating determinants, food choices, motivations, urban setting

## Abstract

Motivation for food choices is one of the most important determinant of eating behavior, because it comes from within the person. The aim of this study was to observe food choice motivations and estimate differences in demographic and health characteristics towards food choice motives in the adult population (*n* = 675; 54% women, ≥18 years) from urban setting. Food choice motivations were assessed using an online questionnaire validated by the EATMOT project. Using K-Means cluster analysis, participants were divided into two clusters of six motivational categories for food choices. Regarding the most and least important motivations, participants in cluster 1 chose food based on emotional motivations, and in cluster 2, they chose based on environmental and political motivations. In addition, younger and obese individuals had more pronounced emotional motivations. In conclusion, this study emphasizes the need to address emotional motivations for healthier food choices among overweight and young people. In addition, the prevalence of health motivations and growing awareness of sustainability indicate a willingness to take actions that benefit personal health and the environment. Apart from providing education, it is society’s responsibility to create an environment that promotes the implementation of acquired knowledge and changes in dietary habits.

## 1. Introduction

Today, is known that a healthy diet can maintain health and protect against the development of the non-communicable diseases, such as obesity, diabetes, cardiovascular disease, cancer, etc. [1]. The World Health Organization (WHO) has issued a Global Action Plan for the Prevention and Control of Noncommunicable Diseases, which consists of several recommendations, one of which emphasizes the role of a healthy diet [2]. WHO points to the need to create national policies and a nutrition-promoting environment, as well as to implement action plans, social marketing initiatives, and public campaigns that promote changes in eating behaviors with the goal of maintaining health. The aims of such nutritional policies include promoting breastfeeding up to six months of age, increasing the consumption of fruit and vegetables, reducing the intake of salt as well as free and added sugars, reducing the intake of saturated fatty acids and trans-fatty acids and replacing them with unsaturated fatty acids, and limiting the consumption of foods with high energy density, which will subsequently limit excessive energy intake [2]. Despite this knowledge, an increasing prevalence of non-communicable diseases can be observed [3]. It was reported that one third (37%) of adults in Croatia suffer from at least one non-communicable disease and that 26% of them are obese. It has also been estimated that 22% of all deaths in 2019 were due to dietary habits, which is more than across the EU [4].

Food choice is a dynamic process that lasts a lifetime. It is estimated that on average, people make a food consumption decision 220 times a day [5,6,7]. According to socio-ecological model theory, food choice is a complex phenomenon influenced by many determinants. At the center of this concept is an individual who has biologically predisposed behaviors such as an innate preference, taste, a sense of satiety, convenience, etc. However, these could be modified by experience and knowledge. Then, various intrapersonal factors such as perception, attitudes, motivation, etc. can influence food choices, as can interpersonal factors (e.g., family, peer, and other social network). Certainly, environmental and social determinants have a major influence on food choices that cannot be ignored. This group of determinants of food choice includes factors such as social relations, cultural practices, social structures, policy, food availability, economic aspects and the information environment, etc. [5,8]. The importance of environmental factors is already evident in the definition of nutrition education, which is a combination of educational strategies and a change in the environment to achieve a change in behavior with the aim of maintaining health [5].

The motivations, one of the interpersonal food choices determinants, are interesting because they come from within the person. The motivation can range from extrinsic to the intrinsic. According to the expectancy-value theories, the individuals measure the outcome of the behavior as beliefs about expected outcome and value of outcome [5,9]. Motivation is an essential component of various theories applied to change eating behavior [5,10]. Therefore, it is necessary to determine the motivations of the population so that nutrition interventions and national actions can be successful. For the assessment of the determinants of food choices, several questionnaires have been most commonly used [11,12,13,14,15,16,17]. However, the novel questionnaire developed within EATMOT project (“Psycho-social motivations associated with food choices and eating practices”) [18] was designed to capture different psychological and social motivations for food choice, as opposed to a particular motivational theory, in different populations and to provide a more comprehensive overview of food choice motivations than existing questionnaires [18,19].

Considering the health problems of the Croatian population [4,20], it is necessary to estimate motivations related to food choices in order to design nutritional policy and implement interventions to change dietary behavior. In Croatia, more than one-third of the population over the age of 18 lives in the five largest cities, and almost one-fifth of the total Croatian population lives in the city of Zagreb, the capital of Croatia [21]. Given this demographic picture, the present study aimed to observe the motivations for food choices among the adult population in an urban setting. It also aimed to determine whether there are differences in food choice motivations among adults and whether they differ by demo-graphic and health characteristics. Consistent with the aims of the study, the following sections describe the questionnaire used to determine the motivations for food choices and the demographic and health characteristics of the study population. Next, the motivations for food choices are presented at the study population level. Finally, the differences in food choice motivations between the two groups within the study population and their common characteristics are presented.

## 2. Materials and Methods

### 2.1. Participants and Settings

The study was cross-sectional and took place within the framework of the international project Psycho-social motivations associated with food choices and eating practices (EATMOT) coordinated by the CI&DETS Research Centre of the Polytechnic Institute of Viseu, Portugal (PROJ/CI&DETS/2016/0008&PROJ/CI&DETS/CGD/0012). In the study, 675 adults (≥18 years old) from the city of Zagreb voluntarily participated. The minimum number of participants of 384 (confidence level of 95%, confidence interval of 5%) ensures the representativeness of the sample of adults from the city of Zagreb (*n* = 633,116; census according to the Croatian Bureau of Statistics from 2011) with regard to participants’ age and sex [21,22]. Data collection was anonymous and no identifiers of participants were collected. Before accessing the questionnaire, participants were given a description of the project and a statement that all information collected would be kept strictly confidential. Completion of the questionnaire was therefore taken as consent to participate. The study was carried out in accordance with the Declaration of Helsinki. The protocols of the study were approved by the Ethics Committee of the Polytechnic Institute of Viseu (registration number: 04/2017) and by the Ethics Committee of the General Hospital of Zadar (number: 01-5623-6|17) for the Croatian population.

### 2.2. Questionnaire and Data Collection

Data collection was through anonymous interviews and online form (Google form). Participants were recruited at the college by email and word of mouth. The validated questionnaire was used to determine the motivation for food choices [18]. Originally, the questionnaire was developed in Portuguese and translated into English, from where it was translated into other native languages of countries within the EATMOT project without making additional changes during the translation process. The questionnaire consisted of 10 sections with closed and opened questions. Section 1 referred to the socio-demographic characteristics of the participants: age, sex, education level, household income, marital status, employment status, work/study sector and whether the participants procured the food themselves. Section 2 included questions on anthropometric characteristics (body weight and height), the level of physical activity, sleep duration, screen time, the frequency of adherence to a healthy diet, whether they follow a specific diet, the presence and type of non-communicable diseases, the presence and type of food allergies or intolerances, and the presence and type of eating disorders. Body mass index (kgm^−2^) was obtained from body weight and height and used to evaluate the weight status of participants according to World Health Organization cut-offs [23]. In order from Section 3 to Section 10, the sections referred to the statements about the following: attitudes relating to health diet; sources of information about healthy diet; health motivations; emotional motivations; economic and availability motivations; social and cultural motivations; environmental and political motivations; and marketing and commercial motivations. In Section 3 and Section 5 through 10, participants indicated how much each statement influenced their food choices on a 5-point scale (1 meaning “strongly disagree” and 5 meaning “strongly agree”). In Section 4 participant indicated the frequency at which they found information about healthy diets (1—never; 5—always). The internal validity of the 49 statements related to the food choices motivations was assessed using Cronbach’s alpha (α = 0.832) [24,25]. For each participant, the six main groups of food choice motivations were expressed as the mean of all statements in each food choice motivation section.

### 2.3. Statistical Analysis

The computer program IBM SPSS Statistics v. 23.0, released in 2015 (IBM SPSS Statistics for Windows, Version 23.0. Armonk, NY, USA: IBM Corp.) was utilized for the data analyzes. Depending on the type of variable, percentages or mean and standard deviation were used to represent the data. The Shapiro–Wilk test was used to determine the continuous data distribution. For the present dataset on food choice motivations the two clusters were proposed by the agglomeration schedule of the Hierarchical cluster analysis with Squared Euclidean distance and Ward’s method. Accordingly, the participants were divided into two clusters of six main groups of food choice motivations using K-Means cluster analysis. The differences in motivation scores between adults in the clusters were analyzed using Student’s *t*-test. The chi-square test for homogeneity was used to estimate differences in adult demographic and health characteristics between two clusters. In all analyses, statistical significance was set at *p* < 0.05.

## 3. Results

The characteristics of the total sample and their health characteristics are shown in Table 1 and Table 2, respectively. Accordingly, of the total study sample, 54.0% were women, while participants were almost evenly distributed across three age categories. Most participants had a university degree (71.4%), and 68.0% were employed at the time of the study. One third of the participants had 4 to 8 h of screen time daily and were moderately physically active. The majority (75.4%) of participants adhered to the basic principles of a healthy diet.

More than 50% of the participants had an adequate weight status (Table 2). One third of the participants had non-communicable diseases, with 7.3% having hypercholesterolemia and 6.2% having hypertension. Of all participants, 13% had a food allergy or intolerance, with most (5.6%) being lactose intolerant. Only 5.8% of the participants had an eating disorder.

**Figure 1:**
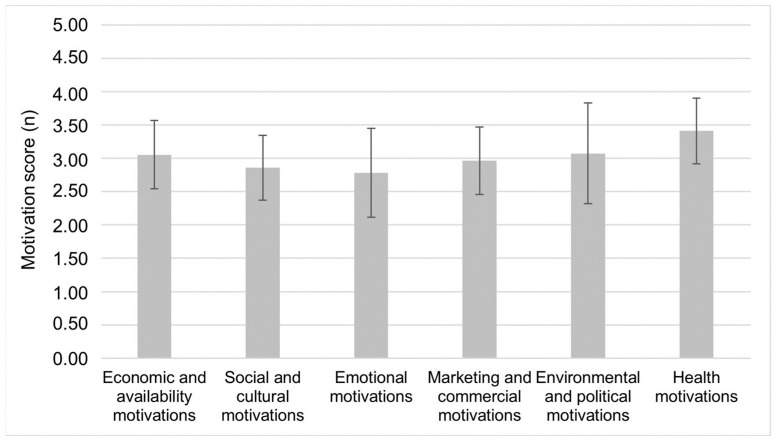
Mean motivation scores for food choices of adults (*n* = 675) from urban setting, rated on a 5-point scale (1 = strongly disagree; 5—strongly agree) using the EATMOT questionnaire. Motivation scores are presented as mean (±standard deviation).

**Figure 2:**
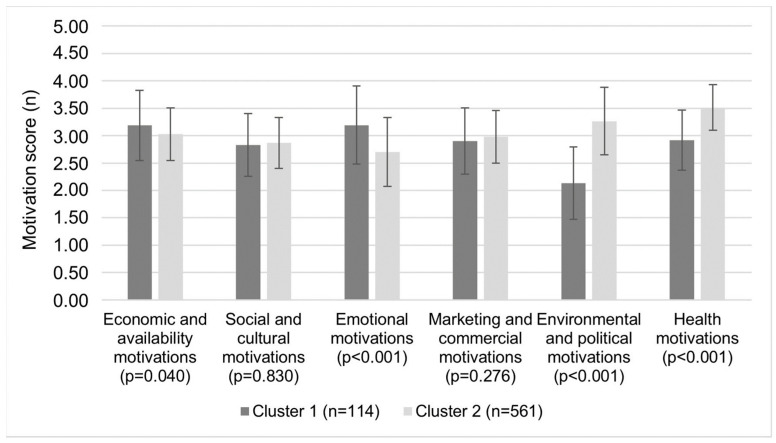
Differences in mean motivation scores for food choices between two clusters of adults from urban setting, rated on a 5-point scale (1 = strongly disagree; 5—strongly agree) using the EATMOT questionnaire. Student’s *t*-test (*p* < 0.05) was used to estimated differences between cluster of adults. Motivation scores are presented as mean (±standard deviation).

In the total sample (Figure 1), the strongest food choice was the health motivations (3.41 ± 0.49), followed by environmental and political motivations (3.07 ± 0.76) and economic and availability motivations (3.05 ± 0.51). Despite the fact that the health motivations for food choices were the strongest in the present study population, the results show that there were two population fractions with respect to the other motivations. Regarding the most important and least important motivations (Figure 2), participants in cluster 1 (16.9% of the total sample) choose food based on emotional motivations (cluster 1: 3.19 ± 0.64; cluster 2: 2.70 ± 0.63; *p* < 0.001), and in cluster 2 (83.1% of the total sample), they chose based on environmental and political motivations (cluster 1: 2.13 ± 0.66; cluster 2: 2.26 ± 0.62; *p* < 0.001). Although significant differences were observed between the clusters in health motivations (2.92 ± 0.55 vs. 3.51 ± 0.42; *p* < 0.001) and economic and availability motivations (3.19 ± 0.64 vs. 3.03 ±. 0.48; *p* = 0.040), both were important motivations in each cluster. The least important motivations and not statistically significant difference in both clusters were marketing and commercial, and social and cultural motivations. If we look at the individual types of motivations (Table 3) in each cluster, participants in cluster 1 choose their food according to their mood, and they associate meals with fellowship and pleasure. In addition, participants in cluster 1 regularly consume foods that they know could have a negative impact on blood sugar and cholesterol levels and choose their food based on price and ease of preparation. Meanwhile, participants in cluster 2 choose the foods they consider part of a balanced diet and are safety to eat. In addition, they pay the most attention to the amount of food they buy in order to avoid food waste. Furthermore, when buying, the participants from the cluster 2 look for foods that are good value for money, such as foods that are on sale, and they prefer to read the nutritional labels.

The demographic and health profiles of participants in each cluster are shown in Table 4. No difference was found between the clusters in terms of sex. The results of the present study show that significantly more (*p* < 0.001) young adults (50%; 18–30 years) belonged to cluster 1, whereas cluster 2 had an even distribution of participants in all three age groups. Regarding health characteristics, significantly more participants in cluster 1 (18.6% vs. 10.2%; *p* < 0.001) were obese than participants in cluster 2. No difference was found in physical activity levels, special diet regime, non-communicable diseases, food allergies or intolerances, or eating disorders.

## 4. Discussion

The results of the present study show the food choice motivations of adults from the urban setting, with health motivation being the strongest. However, there are differences between individuals. For some of the population, emotional motivations tend to dominate alongside health motivations, while for others, environmental and political motivations. Since there are limited data in this area in the Croatian population, the results of this study may be helpful in developing nutrition interventions and communication strategies with consumers.

The present study confirms the results of previous studies, which found that the health motivations was one of the most important motivation among adults from different populations [26,27,28]. The results also confirm that health motivations for food choices predominate among urban adults [28,29]. One of the assumptions for this finding is that the nearly equal distribution of men and women, and people of all ages in this study may reduce the demographic influence on food choice motivations [29]. Second, almost one third of the participants had one of the chronic non-communicable diseases, 33.9% were overweight, and 11.6% were obese, which could increase the health motivations for food choices [26]. Finally, participants in the present study were not evenly distributed by educational level; most had tertiary education. According to the available literature, it seems that adults with higher levels of education have stronger health motivations for their food choices [30], but the results of the EATMOT questionnaires have not been associated with specific motivation group [29]. The results suggest that the adult population urban setting may be willing to change their eating behavior if it improves their health. However, to maintain health motivations, it is necessary to educate people about healthy diet and its effects on the health, accordingly Health Beliefs Model [31,32,33,34]. For such educational efforts to be more effective, the population must have a high level of health literacy [35]. Especially since it is well known that inadequate health literacy is associated with unhealthy eating habits [19]. To the best of the authors’ knowledge, there are no data on the health literacy of the Croatian population. However, it is estimated that 26.6% to 46.3% of the population in the eight European countries have sufficient health literacy and 9.9% to 25.1% have excellent health literacy [35]. Furthermore, the results of the present study confirm previous findings that environmental and political motivations, as well as economic and availability motivations, are the second most important determinants of food choices, albeit with less influence [26,36]. This suggests that adults from urban setting are aware of sustainability and will take actions that benefit the environment. This knowledge can be translated into national policy and campaigns, especially when I know how much the food chain can affect the environment [37].

The results of the cluster analysis from the present study show that 16.9% of the participants belong to the cluster in which the choice of food based on emotional motivations predominates, while environmental and political motivations dominate in 83% of participants. The study with a representative Italian sample showed the dominance of the same three motivations for food choice as in the present study. However, they estimated that environmental and political motivations most strongly influence food choice in the overall population, and the population can be divided into two groups in which health or emotional motivations predominate [38]. This may be because more people in Italy consider themselves healthy, are less overweight or obese, and have fewer chronic non-communicable diseases than in Croatia [4,39]. In addition, the Italian population may be more aware of the importance of a sustainable diet, as this issue has been implemented in the latest dietary guidelines [40]. Accordingly, these may support the need to incorporate new evidence into national food policies and guidelines that support environmental and political motivations for food choices that could impact the maintenance of health and sustainability of the food system. Moreover, the results of the recent study suggest that adults with more dominant health and environmental and political motives are less likely to choose unhealthy foods [19].

Given that emotions can play an important role in food choices, it is not surprising that in the present study population, as in the population of Italian adults, a group of people stands out who make their food choices according to emotional motivations [5,38,41,42]. When observing individual motivations in the present study, participants in cluster 1 choose foods in terms of feelings of comfort. It also seems that participants in the cluster 1 regularly consume foods that they know can have a negative effect on blood sugar and cholesterol levels. This type of food is mainly known as comfort food, and it is known that emotions can overcome the intention of a healthy diet [5,43]. This indicates that in addition to target education, it is necessary to create a healthy environment that promotes the implementation of acquired knowledge. Furthermore, in the present study the participants in cluster 1 associate meals with fellowship and the social environment in which they eat them. This social and cultural motivations is not surprising, when in Croatia, eating cooked meals with family and friends is still more common than consuming fast food [44]. On the other hand, participants in cluster 2 pay attention to the value of food for their money and try to reduce waste when choosing food. These results confirm that participants from the city of Zagreb guided by economics and availability motivations choose food primarily in terms of convenience rather than quality [45], which is more often the case than in other Mediterranean belt countries [26]. Furthermore, the results could be due to the fact that food waste is perceived as a financial loss [46]. This circumstance could be due to the fact that Croatia is one of the poorer countries of the European Union, with an average income that is below the average of the European Union [47].

This study attempts to answer the question of whether participants differ by demographic and health characteristics. No differences were found between clusters in terms of sex in the present study, as Wongprawmas et al. (2021) found no difference in the proportion of participants in terms of sex between health-driven consumers and emotional eating consumers [38]. However, in contrast to the present study, the results of the studies conducted as part of the EATMOT project suggest that women had stronger health, environmental, and political motivations compared to men [29,48]. The results of the present study may be due to the fact that health motivations were strongest in both clusters, and it is known that women are more health-conscious and have a greater motivation for a healthy diet than men [30,49]. Furthermore, age appears to be a variable that influences motivation for food choices [29]. Accordingly, the results of the present study suggest that younger adults tend to choose their foods based on emotional motivations. Indeed, it has been observed in the available literature that emotional motivations were more prevalent in adults younger than 35 years of age [29,38,48,50].

In terms of health characteristics, only body mass index differed between the two clusters, with obese participants having stronger emotional motivations for their food choices. The relationship between weight status and emotional motivations is inconclusive in the available literature. Wongprawmas et al. (2021) found no relationship between weight status and emotional motivations [38], while other findings from previous studies suggest that obese adults are more emotionally motivated in their food choices [26,51,52]. It should be noted that in this study, a smaller proportion of people (11.6%) were obese than estimated in the national sample (26%) [4], and more than half of the study population had an adequate body weight. This raises the question of whether more respondents would report emotional motivations for their food choices if weight status were evenly distributed or if the proportion of obese individuals in the sample were high as in the national sample. According to available literature, adults who do not have non-communicable diseases, food allergies, or eating disorders are most likely to choose foods for economic and availability reasons, in addition to health motives [26], whereas the results of the present study and those of Wongprawmas et al. (2021) suggest no relationship between food choice motivations and non-communicable diseases, food allergies or intolerances, or eating disorders [38]. Furthermore, in the present study, almost the same proportion of participants followed a special diet, which could also be due to the fact that health motivations were the strongest in both clusters, as it has been suggested that health motivations are the main motivation for adults on special diets [26,38]. Although there is no statistically significant difference in the distribution of respondents in terms of physical activity level in the present study, it should be noted that 10% more respondents in cluster 2 had moderate physical activity. According to the Italian sample, adults who were sufficiently physically active had stronger health motivations along with environmental and political motivations than participants who were classified as emotional eating consumers [38]. Namely, in a recent study involving five Mediterranean countries, it was found that participants who were moderately active had the most prominent health motivations for food choice, followed by environmental and political motivations, while other motivations influenced them less [26].

Several limitations must be considered when interpreting the results. The study sample is from the city of Zagreb, and the results are not indicative of the motivations of the entire Croatian population in making food choices. However, Zagreb is the capital city of Croatia, where almost one fifth of the total Croatian population over 18 years of age lives [21]. In addition, the sample size was representative of the population of the city of Zagreb and included almost equal numbers of men and women in all three age groups. Body mass index was calculated from the measurements obtained from the participants, and no anthropometric measurement was performed. However, it has been suggested that self-reported height and weight may be used in epidemiologic studies with a representative sample of the study population [53].

## 5. Conclusions

There is an overlap of motives for food choices, and there is not just one motivation that could determine food choices among individuals. Among adults from the urban setting, health motivations were most prominent, while there were differences in emotional, environmental, and political motivations. Younger and obese individuals exhibited more pronounced emotional motivations, meaning that they choose foods based on their feelings and regularly consciously consume foods that may have negative health consequences. The results of this study suggest that intervention in obese and young people’s emotional motivations is needed to help them make better food choices and adopt healthier eating habits. In addition, it would be desirable to apply the Health Belief Model in the interventions and link the influence of diet on health, as health motivations for food choices dominated in the study group. Moreover, the large number of respondents who have a more pronounced environmental and political motivations in their food choices, in addition to the health motivations, suggests that people are becoming more aware of the unsustainability of the current food system and that they can influence it through their behavior. This finding suggests that people are ready for interventions that lead to changes in eating behavior that not only maintain their health but also contribute to the environment. Aside from delivering education, there is a broader social responsibility to create an environment supportive of the implementation of acquired knowledge and fostering dietary habit changes. This responsibility encompasses not only the dissemination of information but also the creation of a supportive framework wherein individuals are empowered and motivated to translate knowledge into tangible and sustained improvements in their eating behaviors.

## Figures and Tables

**Table 1:** Demographic characteristics and lifestyle of adults from urban setting in the total sample ^1^.

Characteristics	Total Sample (*n* = 675)
Sex	
Male	45.9%
Female	54.1%
Age	
18–30 years	35.1%
31–50 years	33.8%
51–85 years	31.1%
Education level	
Elementary school	0.6%
High school	28.0%
Faculty	71.4%
Employment	
Student	15.1%
Student with employment	4.7%
Unemployed	4.6%
Employed	68.0%
Retired	7.6%
Screen time	
0–2 h	23.1%
2–4 h	21.9%
4–8 h	31.1%
>8 h	22.8%
Unknown	1.0%
Physical activity level	
Never	8.4%
Rarely	21.5%
Occasionally	25.0%
Moderate	31.0%
Intensive	14.1%
Specific nutrition	
Raw food	0.4%
Vegetarian	1.5%
Vegan	0.6%
Flexitarian	4.6%
Restriction of energy intake	6.4%
Religion	1.6%
Other	6.5%
Principal of healthy diet	75.4%
Unknown	2.9%

^1^ All variables are presented as percentages.

**Table 2:** Health characteristics of adults from urban setting in the total sample ^1^.

Characteristics	Total Sample (*n* = 675)
Body mass index	
<18.5 kgm^−2^	2.5%
18.5–24.9 kgm^−2^	51.9%
25.0–29.9 kgm^−2^	33.9%
>30.0 kgm^−2^	11.6%
Unknown	0.1%
Non-communicable disease	
Diabetes	4.0%
Hypercholesterolemia	7.3%
Hypertension	6.2%
Gastritis	3.3%
Bowel disease	0.7%
Obesity	4.0%
Other	6.5%
None	68.0%
Food allergy/intolerance	
Lactose	5.6%
Casein	0.1%
Gluten	2.5%
Nuts	2.1%
Shellfish	0.6%
Other	4.6%
None	87.0%
Eating disorder	
Bulimia	0.4%
Anorexia	0.9%
Compulsive overeating	3.7%
Other	0.6%
None	94.2%
Bulimia	0.4%

^1^ All variables are presented as percentages.

**Table 3:** The 10 most dominant food choice motivations assessed using the EATMOT questionnaire each cluster of adults from urban setting ^1^.

Food Choice Motivations Category	Statement from the EATMOT Questionnaire	Score
**Cluster 1 (*n* = 114)**		
Economic and availability motivations	I choose the food I consume because it isconvenient to purchase.	3.72 ± 0.94
Emotional motivations	Food makes me feel good.	3.65 ± 0.94
Social and cultural motivations	Meals are time of fellowship and pleasure.	3.61 ± 0.98
Emotional motivations	I eat more when I have nothing to do.	3.60 ± 1.09
Economic and availability motivations	I usually buy food that is easy to prepare.	3.53 ± 1.00
Economic and availability motivations	I usually choose food that had a good quality/price ratio.	3.47 ± 1.02
Health motivations	There are some foods that I consume regularly, even if they may raise my blood glycaemia.	3.39 ± 1.08
Economic and availability motivations	I buy fresh vegetables to cook myself moreoften than frozen.	3.37 ± 1.12
Health motivations	There are some foods that I consume regularly, even if they may raise my cholesterol.	3.34 ± 1.05
Emotional motivations	Food helps me cope with stress.	3.30 ± 1.24
**Cluster 2 (*n* = 561)**		
Health motivations	It is important for me to eat food that keeps me healthy.	4.01 ± 0.72
Environment and political motivations	When I cook, I have in mind the quantitiesto avoid food waste.	3.92 ± 0.81
Health motivations	I am very concerned about the hygiene andsafety of the food I eat.	3.83 ± 0.87
Health motivations	It is important for me that my daily diet contains a lot of vitamins and minerals.	3.80 ± 0.76
Economic and availability motivations	I usually choose food that had a good quality/price ratio.	3.79 ± 0.80
Marketing and commercial motivations	When I go shopping, I prefer to read food labels instead of believing in the advertising campaigns.	3.77 ± 0.98
Economic and availability motivations	I buy fresh vegetables to cook myselfmore often than frozen.	3.75 ± 0.94
Health motivations	Usually I follow a healthy and balanced diet.	3.68 ± 0.78
Economic and availability motivations	I usually buy food that it is on sale.	3.66 ± 0.87
Health motivations	I avoid food with genetically modified organisms.	3.65 ± 1.10

^1^ Motivation scores were rated on a 5-point scale (1 = strongly disagree; 5—strongly agree) and presented as mean (±standard deviation).

**Table 4:** Comparison of demographic and health characteristics of the adults from urban setting between clusters ^1^.

Characteristics	Cluster 1 (*n* = 114)	Cluster 2 (*n* = 561)	*p* Values *
Sex			
Male	53.5%	44.4%	0.075
Female	46.6%	55.6%	
Age			
18–30 years	50.0%	32.1%	<0.001
31–50 years	32.5%	34.0%	
51–85 years	17.5%	33.9%	
Physical activity			
Never	14.0%	7.3%	0.066
Rarely	24.6%	20.9%	
Occasionally	23.7%	25.3%	
Moderate	22.8%	32.6%	
Intensive	14.9%	13.9%	
Specific nutrition			
Yes	23.1%	22.8%	0.942
No	76.9%	77.2%	
Body mass index			
<18.5 kgm^−2^	2.7%	2.5%	0.036
18.5–24.9 kgm^−2^	53.1%	51.7%	
25.0–29.9 kgm^−2^	25.7%	35.7%	
>30.0 kgm^−2^	18.6%	10.2%	
Non-communicable disease			
Yes	6.1%	4.1%	0.335
No	93.9%	95.9%	
Food allergy/intolerance			
Yes	18.4%	11.9%	0.061
No	81.6%	88.1%	
Food disorder			
Yes	6.1%	5.5%	0.799
No	93.9%	94.5%	

^1^ All variables are presented as percentages. * Chi-square test (*p* < 0.05) was used to estimate the differences in characteristics of the adults between two clusters.

## Data Availability

The data that support the findings of this study are available from the corresponding author, I.R., upon reasonable request.
